# Assessment of infarct characteristics and left ventricular function on serial CMR in STEMI patients treated with post-PCI sonothrombolysis: post-hoc analysis of two randomized controlled trials

**DOI:** 10.1016/j.ijcha.2025.101757

**Published:** 2025-07-23

**Authors:** Soufiane El Kadi, Shouqiang Li, Chad Hovseth, Luuk H.G.A. Hopman, Mariëlle C. van de Veerdonk, Niels J.W. Verouden, Feng Xie, Albert C. van Rossum, Otto Kamp, Thomas R. Porter

**Affiliations:** a Amsterdam UMC, Location VUmc, Cardiology, Amsterdam Cardiovascular Sciences, Amsterdam, the Netherlands; b University of Nebraska Medical Center, Division of Cardiovascular Medicine, Omaha, NE, United States

**Keywords:** STEMI, Sonothrombolysis, Cardiovascular magnetic resonance, Systolic function, Infarct pattern

## Abstract

**Background:**

Several randomized clinical trials have studied sonothrombolysis as adjunctive treatment in ST-elevation myocardial infarction (STEMI) patients to reduce infarct size (IS) and preserve left ventricular (LV) function. This study aims to assess infarct characteristics and LV function in STEMI patients treated with sonothrombolysis following primary percutaneous coronary intervention (PCI) on cardiovascular magnetic resonance (CMR) imaging..

**Methods:**

Fifty-two STEMI patients were prospectively randomized to receive sonothrombolysis immediately following PCI and underwent early (within seven days after STEMI) and follow-up (6–8 weeks) CMR imaging. IS and distribution pattern, microvascular obstruction, intramyocardial hemorrhage and T1/T2-mapping of infarct and remote zone, as well as LV global longitudinal strain (GLS) and LV ejection fraction (LVEF) were assessed on early CMR. IS and LV systolic function were also assessed on follow-up CMR.

**Results:**

Mean age was 58 years, and culprit artery was predominately left anterior descending artery in both groups (92 % and 93 %, respectively). Although there were no differences in IS at baseline and follow-up, infarct pattern was significantly different between the groups on early CMR (patchy LGE pattern in 46 % of the sonothrombolysis vs. 19 % control group, p = 0.04). Significant LVEF improvement (ΔLVEF:7.2 ± 5.4 %, p < 0.01 vs ΔLVEF: 0.9 ± 7.2 %, p = 0.29) and GLS improvement (|ΔGLS|: 3.2 ± 3.2 %, p < 0.01 vs. |ΔGLS|:1.5 ± 4.2 %, p = 0.07) was observed in the sonothrombolysis group, but not in the control group.

**Conclusion:**

LV systolic function improvement at 6–8 weeks following STEMI was observed in patients treated with post-PCI sonothrombolysis independent of IS reduction. Further investigation into the effects of post-PCI sonothrombolysis on infarct zone viability is needed.

## Graphical abstract

**Figure:**
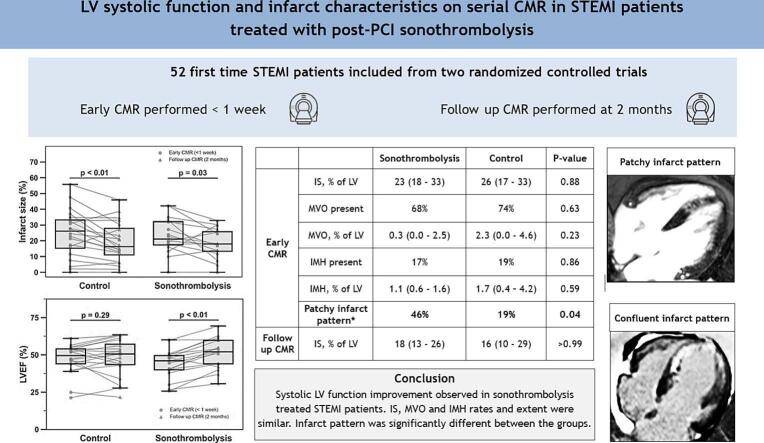

## 1 Background

Despite significant advancements in the treatment of ST-elevation myocardial infarction (STEMI), myocardial infarction remains a leading cause of morbidity and mortality globally. One of the major challenges in contemporary management of STEMI is minimizing ischemic/reperfusion injury and microvascular obstruction (MVO) following primary percutaneous coronary intervention (PCI) [1]. Following PCI for STEMI, a complex cascade of consecutive events take place that co-determine the extent of the final infarct and the degree of adverse remodeling [2]. These events include but are not limited to intra- and extracellular edema as a consequence of inflammation, distal embolization, in-situ thrombosis and plugging causing MVO and hemorrhage due to irreversibly damaged endothelium. Using cardiovascular magnetic resonance (CMR) imaging, myocardial infarct size (IS) and MVO can be characterized in detail using late-gadolinium enhancement (LGE), while parametric mapping techniques and T2-weighted imaging offer the ability to assess for edema and hemorrhage [3]. Furthermore, CMR provides a more quantitative and independent imaging method to analyze regional and global systolic function. Sonothrombolysis, the adjunctive use of ultrasound and intravenously infused microbubbles, has emerged as a promising therapeutic modality aimed at increasing thrombolysis and enhancing microvascular reperfusion in cardiovascular ischemic diseases [4]. Although pre-clinical research and initial clinical studies suggested potential benefits of sonothrombolysis in improving myocardial perfusion and function in STEMI patients [[5], [6], [7], [8]], recent investigations have yielded conflicting results regarding its efficacy in attenuating infarct size [[9], [10], [11]]. An improved understanding of early effects of sonothrombolysis on the myocardium at risk and surrounding myocardial tissue is pivotal for determining the value and underlying mechanisms of a potential beneficial effect on systolic function. Further, as sonothrombolysis could also lead to increased temporary permeability of vascular endothelium, it is important to determine whether or not sonothrombolysis is associated with increased intramyocardial hemorrhage (IMH). This manuscript aims to comprehensively investigate the differential effects of sonothrombolysis on myocardial tissue composition in the infarct and remote zone and systolic function in STEMI patients treated with sonothrombolysis post-PCI.

## 2 Methods

In this study, STEMI patients from two clinical trials [9,11] who, as part of the study protocol, were prospectively randomized to sonothrombolysis or control following PCI and who underwent CMR imaging within seven days after STEMI, were included. Patients with previous myocardial infarction were excluded. Both trials were approved by the local medical ethics committee and conducted according to the guidelines for Good Clinical Practice, and complied with the Declaration of Helsinki. All patients provided informed consent before participation in the trials.

### 2.1 Study procedures

Patients with non-anterior STEMI who had incomplete ST-resolution (< 70 %) after PCI, and patients with anterior STEMI (both groups with symptoms < 12 h) were randomly allocated (1:1) to either adjunctive sonothrombolysis or control. Both groups received a continuous infusion of 3 % Luminity microbubbles (LUMINITY®, Lantheus Medical Imaging, Inc., North Billerica, MA) with an infusion rate of 2–4 mL/minute. The sonothrombolysis group received simultaneous low mechanical index (LMI) ultrasound (MI < 0.2) with 40–90 intermittent high mechanical index (HMI) pulses (MI 1.2; pulse duration < 5 µs, flash frames 10, transmit-receive frequency 1.8 MHz/1.8 MHz, focus at mitral valve) using the Philips iE-33 machine or EPIQ and S5-1 or X5-1 transducer (Andover, MA). HMI pulses were given from the apical windows with in-between tilting and rotating of the transducer to cover all of the myocardium. Patients allocated to the control group received continuous LMI ultrasound (MI < 0.2), with no more than six HMI pulses in total, intended for perfusion imaging.

### 2.2 CMR protocol

Within two to seven days post-infarction, CMR imaging was performed with a 1.5 or 3.0-T clinical scanner (Siemens Healthcare, Erlangen, Germany, and Philips Achieva Plat-form, Eindhoven, Netherlands). Long and short-axis cine images were obtained using a balanced steady-state free-precession sequence to assess left ventricular volume, mass and function. T2-weighted turbo spin-echo sequences with fat saturation images were obtained in short-axis and long-axis views to cover the left ventricle (slice thickness 8 mm, inter-slice gap 2 mm, normalization filter on). Through the infarct core, native T1-mapping was performed using the Shortened Modified Look-Locker Inversion Recovery (ShMOLLI) technique, along with T2 mapping. LGE images were acquired 10–15 min after administration of routine gadolinium-based contrast (Dotarem, Guerbet; 0.2 mmol/kg) using a 2-dimensional segmented inversion recovery gradient-echo pulse sequence with identical slice position as the cine images. Measurements were performed using Circle CVI^42^ (version 5.13, Circle Cardiovascular Imaging, Inc., Calgary, AB, Canada) by experienced CMR readers blinded to randomization. Left ventricular end-diastolic volume (LVEDV) and mass, alongside end-systolic volume (LVESV) and left ventricular ejection fraction (LVEF), were derived from the short-axis cine images by manually delineating the endocardial and epicardial borders during both end-diastolic and end-systolic phases.

Global longitudinal strain (GLS) was analyzed from three long-axis cine views using CVI42′s feature-tracking module. Endocardial contours were manually drawn at end-diastole and automatically propagated throughout the cardiac cycle. GLS was reported as the average peak systolic strain from all long-axis views and expressed as a negative percentage, with more negative values indicating better systolic function.

**Fig. 1:**
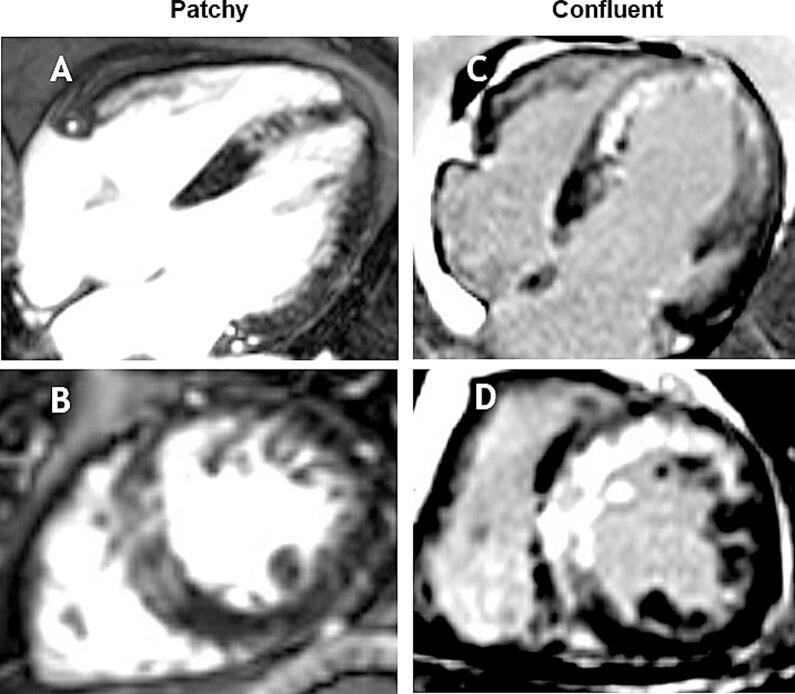
Example assessment LGE pattern*.* A, B: CMR-LGE acquisition of the AP4CH view and SAX view demonstrating patchy infarction. C,D: CMR-LGE acquisition of the AP4CH view and SAX view demonstrating confluent infarction. Abbreviations: AP4CH = apical 4-chamber view; CMR = cardiovascular magnetic resonance; LGE = late gadolinium enhancement; SAX = short axis.

Edema quantification was conducted using short-axis T2-weighted images, and expressed as a percentage of LV mass. T1 and T2 values were calculated based on drawings of regions of interest in the infarct zone (outside the zone of microvascular obstruction) and remote zone, and normalized by dividing the values by local scanner- and population specific reference values. IS measured on short-axis LGE images, was quantified using the full-width at half-maximum method, and reported in grams and as a percentage of LV mass [12]. Transmurality of the infarct was defined as the number of segments with > 50 % of the circumferential length of the segment displaying > 75 % transmural damage as demonstrated by LGE. Edema was defined as voxels exhibiting a signal intensity exceeding two standard deviations above that of remote myocardium. IMH was discerned on T2-weighted images by the presence of a dark core within hyper intense area of edema [13]. MVO was identified as hypo-intense cores within hyper enhanced myocardium on LGE images and were included for calculation of IS. Endocardial extent of LGE was assessed by summing tracings of endocardial enhancement on all short-axis LGE images and dividing by total summation of all endocardial contours. Pattern of LGE was scored in a dichotomic fashion as either confluent or patchy on early and follow up CMR (Fig. 1). Classic MVO pattern consistent with aforementioned description was considered confluent. In equivocal cases, simultaneous assessment of cine loops of corresponding slices was used to determine LGE pattern, i.e. confluent when akinesis of the infarct area was observed, or patchy in case of hypo-/normokinesis of the infarct area. Follow-up CMR was performed 6–8 weeks after the index infarction to reassess infarct size, MVO presence, endocardial extent of LGE, transmurality of myocardial damage, infarct morphology, and myocardial function.

### 2.3 Statistical analysis

Continuous variables were presented as either mean with standard deviation or median with interquartile range (IQR), whereas categorical variables were presented as percentages. To compare continuous data between the intervention and control groups, independent-samples t-tests were used for normally distributed variables, and Mann-Whitney U tests were used for non-normally distributed variables. Paired t-tests were used for repeated measurements. Fisher’s exact test or χ^2^ test was utilized to compare dichotomous and categorical data. Statistical significance was determined with a two-sided p-value of less than 0.05. For repeated measurements with an expected directional hypothesis, a one-sided p-value of less than 0.05 was considered significant. No correction was performed for multiple testing.

## 3 Results

**Fig. 2:**
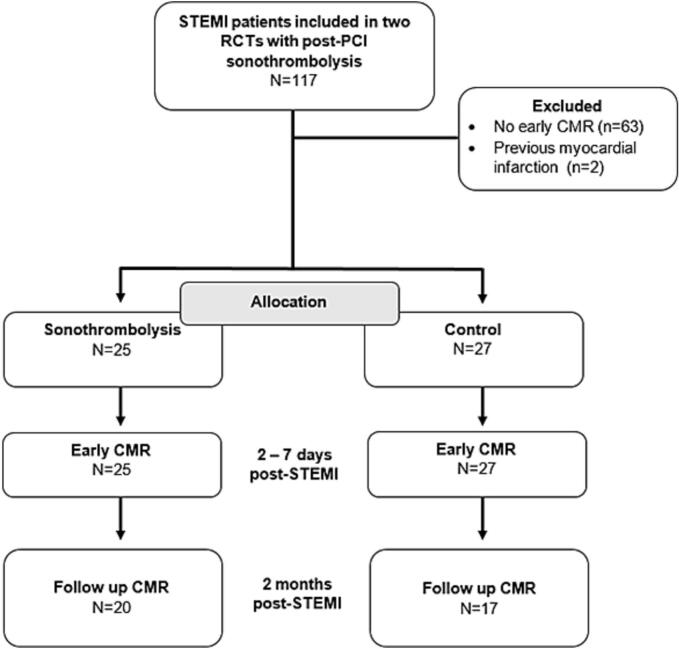
Inclusion chart. Abbreviations: CMR = cardiovascular magnetic resonance; PCI = percutaneous coronary intervention; RCT = randomized controlled trial; STEMI = ST-elevation myocardial infarction.

Between 2017 and 2023, a total of 117 patients were included in the two randomized clinical trials, of whom 52 (44 %) underwent early CMR (25 randomized to sonothrombolysis, 27 randomized to control, Fig. 2). The baseline characteristics were balanced between groups. Mean age was 58 years, with a majority of males in both groups. The body mass index, systolic blood pressure, and rates of diabetes, dyslipidemia, and smoking were similar. Hypertension was more prevalent in the control group. Medication use prior to admission and timing metrics were comparable. Regarding angiographic characteristics, the majority of patients presented with one-vessel disease, with similar distribution between the sonothrombolysis and control groups (56 % vs. 63 %, respectively). The culprit artery was predominantly the left anterior descending artery LAD in both groups (92 % and 93 %, respectively). Pre-PCI Thrombolysis in Myocardial Infarction (TIMI) flow grades showed comparable distributions. Post-PCI TIMI flow grades also demonstrated similarities between the groups, with the majority achieving TIMI 3 flow post-procedure (72 % and 70 % for sonothrombolysis and control group, respectively). Post-procedural TIMI flow < 3, indicative of slow- or no-reflow, was observed in 28 % of patients in the sonothrombolysis group and 30 % in the control group. Detailed baseline characteristics are provided in Table 1.

**Table 1:** Baseline characteristics.

		Sonothrombolysis (n = 25)	Control (n = 27)
Age, y		58 (±8)	58 (±10)
Male sex, n (%)		19 (76)	23 (85)
Body mass index, kg/m^2^		30 (27–31)	29 (24–34)
Systolic blood pressure, mmHg		136 (129–148)	131 (112–153)

Medical history, n (%)			
Diabetes mellitus		5 (18)	4 (13)
Hypertension		8 (32)	13 (48)
Dyslipidemia		17 (68)	18 (67)
Smoking		11 (44)	7 (26)
Positive family history		17 (68)	15 (56)

Drug use before admission			
Aspirin		3 (12)	3 (11)
Statin (Lipid lowering drug use)		5 (20)	3 (11)
Beta-blocker		1 (4)	3 (11)
Calcium-channel blocker		3 (12)	3 (12)
ACE-i / ARB		3 (12)	6 (22)

Timing			
Symptom-to-diagnosis time (min)		88 (39–124)	106 (44–373)
Diagnosis-to-reperfusion time (min)		50 (37–65)	43 (35–59)

**Angiographic characteristics**			
Atherosclerotic burden	One-vessel	14 (56)	17 (63)
	Two-vessel	7 (28)	6 (22)
	Three-vessel	4 (16)	4 (15)
Culprit artery	LAD	23 (92)	25 (93)
	LCx	0 (0)	0 (0)
	RCA	2 (8)	2 (7)
Pre-procedural TIMI flow	0	16 (64)	18 (67)
	I	1 (4)	3 (11)
	II	7 (28)	2 (7)
	III	1 (4)	4 (15)
Post-procedural TIMI flow	0	0 (0)	0 (0)
	I	0 (0)	0 (0)
	II	7 (28)	8 (30)
	III	18 (72)	19 (70)

Values are depicted as mean ± standard deviation, median (interquartile range) or number (percent).

Post-procedural TIMI flow < 3 was considered indicative of slow- or no-reflow phenomenon.

Abbreviations: ACE-i: angiotensin-converting enzyme inhibitor; ARB: angiotensin-II receptor blocker; PCI: percutaneous coronary intervention; SBP: systolic blood pressure.

### 3.1 Infarct characteristics

**Fig. 3:**
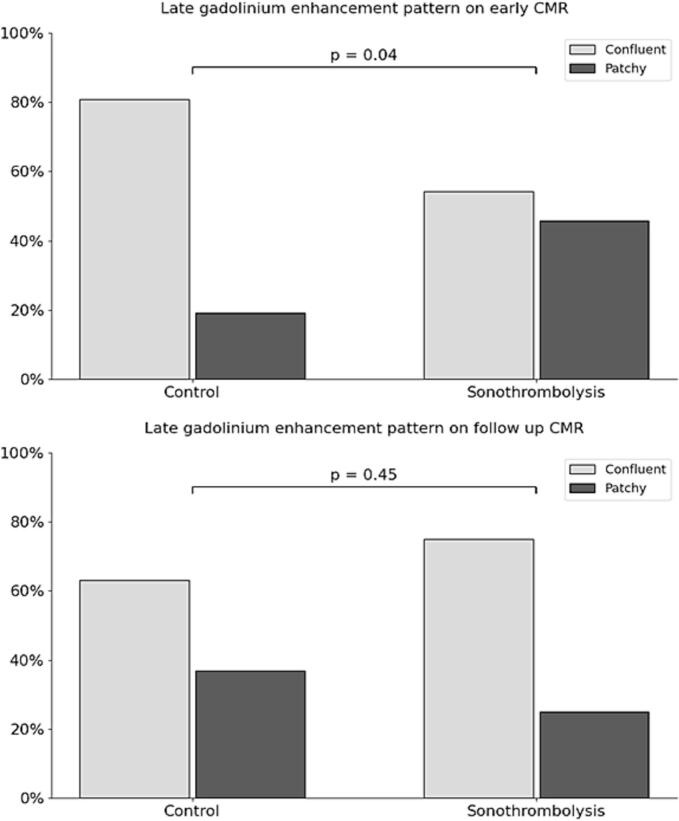
LGE pattern on early and late CMR. Abbreviations: CMR = cardiovascular magnetic resonance; LGE = late gadolinium enhancement.

IS in grams (sonothrombolysis 30 g [IQR: 20 – 68], control 34 g [IQR: 25 – 49], p = 0.83) and relative to LV mass (sonothrombolysis 23 % [IQR: 18 – 33], control 26 % [IQR: 17 – 33], p = 0.88) were comparable on early CMR (Table 2). MVO, intramyocardial hemorrhage and edema had a similar frequency in both groups and were similar in size. MVO on the initial CMR was observed in > 70 % of all patients, while IMH was detected in less than 20 % of the total cohort. In patients with IMH, size of the hemorrhage was not different in the sonothrombolysis group versus the control group (sonothrombolysis 2.1 g [IQR: 1.4 – 2.8], control 2.7 g [IQR: 1.0 – 2.8], p > 0.99). Normalized T1 values in the infarct and remote zone were also not different, however, normalized T2 value in the remote zone tended to be lower in the sonothrombolysis group (sonothrombolysis 0.91 [IQR: 0.91 – 0.91], control 0.98 [IQR: 0.95 – 1.01], p = 0.05). Delayed enhancement patterns were significantly different between the groups on early CMR imaging (Fig. 3). Patchy LGE patterns were observed in 46 % of the sonothrombolysis treated groups vs. 19 % of the control group (χ^2^ = 4.1, p = 0.04). On follow up CMR scans, no difference in LGE pattern was observed (patchy LGE in 25 % in sonothrombolysis vs. 37 % in control, p = 0.45). Also IS at this time point was similar in both groups as was the number of segments with transmural myocardial damage.

**Table 2:** Infarct characteristics.

		Sonothrombolysis	Control	p-value
Early CMR	Time to early CMR, days	3 (2 – 4)	2 (1.5 – 3)	0.18
	IS, grams	30 (20–68)	34 (25–49)	0.83
	IS, %	23 (18–33)	26 (17–33)	0.88
	Endocardial LGE, %	34 (29 – 43)	35 (29–41)	0.96
	Transmurality of myocardial damage, # segments	5.0 (4.0–8.0)	6.0 (5.0 – 8.0)	0.92
	MVO present, n (%)	17 (68)	20 (74)	0.63
	MVO, grams	0.7 (0.0–5.4)	2.8 (0.0 – 7.2)	0.35
	MVO, %	0.3 (0.0–2.5)	2.3 (0.0–4.6)	0.23
	IMH present, n (%)	4 (17)	5 (19)	0.86
	IMH, grams	2.1 (1.4–2.8)	2.7 (1.0–2.8)	>0.99
	IMH, %	1.1 (0.6–1.6)	1.7 (0.4 – 4.2)	0.59
	Edema present, n (%)	20 (83)	19 (70)	0.28
	Edema, %	28 (16–33)	31 (16–43)	0.72
	AAR, grams	23 (17–59)	33 (21–59)	0.67
	T1 infarct zone, normalized	1.20 (1.16–1.24)	1.19 (1.16–1.26)	0.84
	T1 remote zone, normalized	1.04 (1.00–1.05)	1.03 (1.00–1.07)	0.92
	T2 infarct zone, normalized	1.35 (1.35–1.39)	1.31 (1.28–1.35)	0.29
	T2 remote zone, normalized	0.91 (0.91–0.91)	0.98 (0.95–1.01)	0.05
Follow up CMR	Time to follow up CMR, days	47 (45 – 60)	54 (45 – 63)	0.46
	IS, grams	20 (12–41)	22 (10–32)	0.79
	IS, %	18 (13–26)	16 (10–29)	>0.99
	Endocardial LGE, %	28 (22–35)	28 (17–39)	0.94
	MVO present, n (%)	4 (25)	8 (40)	0.34
	Transmurality of myocardial damage, # segments	5.0 (3.0–7.0)	5.5 (1.5–7.5)	0.99

Values are depicted as median (interquartile range) or number (percent).

Abbreviations: AAR = area at risk; CMR = cardiac magnetic resonance; IMH = intramyocardial hemorrhage; IS = infarct size; LGE = late gadolinium enhancement; MVO = microvascular obstruction.

### 3.2 LV volume and function

No significant differences were observed between the sonothrombolysis and control group regarding absolute volumetric cardiac indices on early and late CMR studies (Table 3). At the group level, LVEF measured on early CMR (sonothrombolysis 43 % [IQR: 38–49], control 47 % [IQR: 39–54], p = 0.27) and on late CMR (sonothrombolysis 52 % [IQR: 44–60], control 51 % [IQR: 43–58], p = 0.59) were not statistically different. GLS was also similar on group level.

**Table 3:** Left ventricular volumes and function.

		Sonothrombolysis	Control	p-value
Early CMR	LV mass, grams	167 (126–199)	137 (130–167)	0.22
	LVEDV, mL	173 (149–206)	174 (143–206)	0.83
	LVEDVi, ml/m^2^	84 (69–97)	78 (65–92)	0.58
	LVESV, mL	97 (79–124)	91 (72–131)	0.52
	LVESVi, ml/m^2^	46 (39–61)	39 (35–52)	0.25
	LVEF, %	43 (38–49)	47 (39 – 54)	0.27
	GLS, %	−9 (−11 − −7)	−11 (−12 − −8)	0.34
Follow up CMR	LV mass, grams	128 (111 – 158)	121 (105–140)	0.41
	LVEDV, mL	184 (140 – 226)	166 (150–209)	0.60
	LVEDVi, ml/m^2^	90 (71–100)	76 (70–91)	0.27
	LVESV, mL	97 (73–108)	79 (65–141)	0.86
	LVESVi, ml/m^2^	44 (34–54)	37 (30–52)	0.50
	LVEF, %	52 (44 – 60)	51 (43–58)	0.59
	GLS, %	−12 (−14 − −10)	−12 (−14 − −9)	0.82

Values are depicted as median (interquartile range).

Abbreviations: CMR = cardiac magnetic resonance; GLS = global longitudinal strain; LVEF = left ventricular ejection fraction; LVEDV = left ventricular end-diastolic volume; LVEDVi = left ventricular end-diastolic volume index; LVESV = left ventricular end-systolic volume; LVESVi = left ventricular end-systolic volume index; LV mass = left ventricular mass.

### 3.3 Change in IS and LV function

**Fig. 4:**
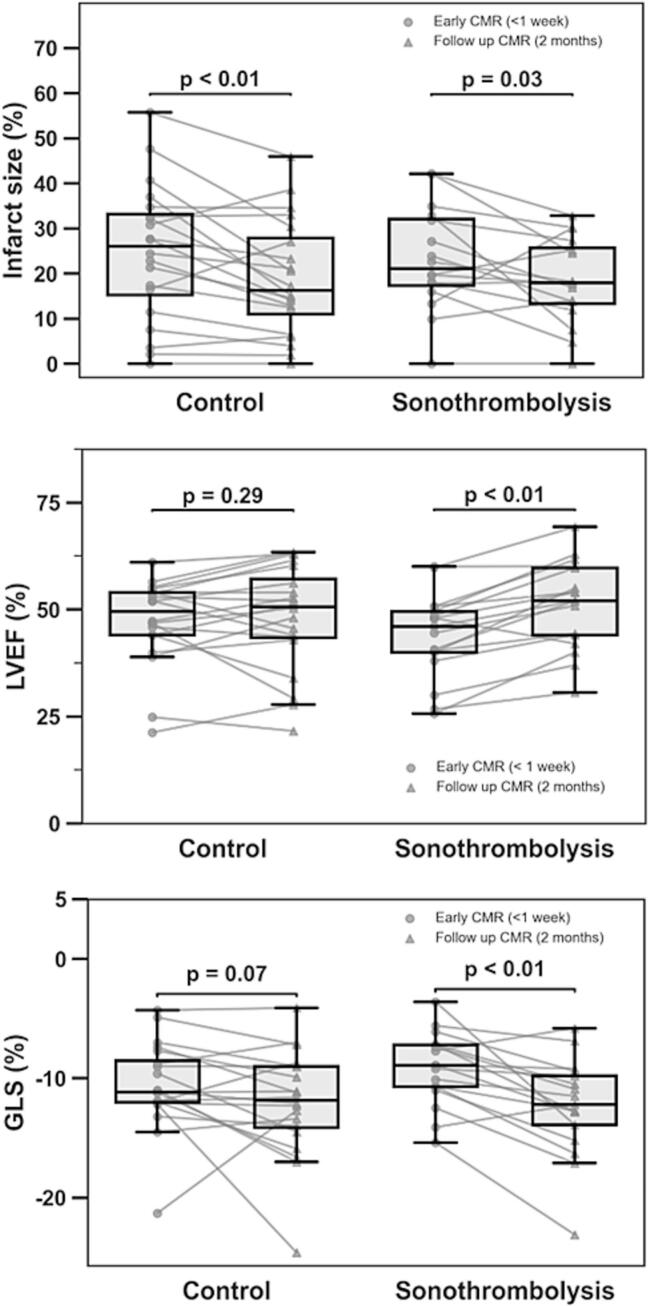
Change in infarct size and LV systolic function per group. Abbreviations: CMR = cardiovascular magnetic resonance; GLS = global longitudinal strain; LVEF = left ventricular ejection fraction.

Percentual changes in IS, LVEF and GLS between early and late CMR studies were different (Fig. 4). Statistically significant IS reduction was observed in both groups (mean Δ IS: sonothrombolysis −4.9 ± 9.7, p = 0.03 and control −5.7 ± 8.7, p < 0.01). LVEF increased in the sonothrombolysis group (mean ΔLVEF: 7.2 ± 5.4, p < 0.01), but not in the control group (mean ΔLVEF: 0.9 ± 7.2, p = 0.29). GLS improved significantly in the sonothrombolysis group (|ΔGLS|: 3.2 ± 3.2, p < 0.01) but not in the control group (|ΔGLS|: 1.5 ± 4.2, p = 0.07).

## 4 Discussion

This is the first study assessing in detail the infarct features and myocardial tissue characteristics of STEMI patients treated with post-PCI sonothrombolysis using serial CMR imaging. IS was relatively large early after STEMI (∼25 % of LV mass), decreasing only minimally after two months. No differences between sonothrombolysis and control were observed in well-established prognostic CMR parameters, in particular not in MVO and IMH. Using T2W images, IMH was seen in less than 20 % of the current cohort. Although the incidence in this study corresponds to numbers reported in earlier trials [14,15], it is less than what might be expected in a STEMI population where MVO was present in 70 % of patients. Notably, IMH was not more prevalent in the sonothrombolysis group compared to the control group, reassuring that currently used sonothrombolysis protocols are safe in terms of myocardial bleeding. Native T1 and T2 mapping are informing of inflammation and are associated with LV remodeling [[16], [17], [18], [19]]. Due to the use of different scanners, normalized T1 and T2 values were calculated in the infarct and remote zone. In line with literature, T1 and T2 values in the infarct zone were higher in both groups, without statistically significant between-group differences. Normalized T2 values in the remote zone were slightly lower in the sonothrombolysis group compared with the control group, which may reflect reduction of inflammation and thrombosis due to cavitation-mediated increase in microvascular perfusion [20,21]. The increase in GLS and LVEF between early and follow up CMR scans was significant only in the sonothrombolysis group. Since IS was comparable between the groups at baseline and follow up, the increased systolic LV function in the sonothrombolysis arm could be explained by preserved regional function of the remote myocardium. Another cause of the observed improvement of LV function despite similar IS could be more diffuse and heterogenous infarct zones in the sonothrombolysis patients, with intermingled viable strands of myocardium that may contribute to systolic function. Patchy enhancement pattern was indeed seen more frequently in the sonothrombolysis group on early CMR, while endocardial and transmural degree of LGE were comparable between the groups. Currently, it is unclear whether a patchy LGE pattern is associated with improved systolic recovery. Additionally, it is challenging to distinguish viable myocardium within the infarct area from MVO, although the latter is usually characterized by a dark hypo-intense core within a dense area of LGE and does not display viability on cine loops. Further research into infarct pattern and distribution and regional myocardial dynamics in the infarct area and remote zone in patients treated with sonothrombolysis is warranted. Of interest, an ongoing study on pre- and post-PCI sonothrombolysis in STEMI patients will shed light on the immediate effects of sonothrombolysis on coronary physiology by invasively measuring indices of microvascular resistance [22].

### 4.1 Limitations

There are several limitations to the current study. First, this is a post-hoc analysis. The analyses were not pre-specified and the results of these study should therefore be considered hypothesis-generating. Second, a significant portion of the patients of the original trials were excluded due to the lack of early CMR imaging, which may have affected the results. Furthermore, detailed data on periprocedural pharmacologic agents that could affect infarct size and morphology were not available; however, the standard protocol in both participating centers discourages the routine use of adjunctive therapies such as GP IIb/IIIa inhibitors or intracoronary vasodilators during primary PCI, which limits potential variability from these agents. Different CMR scanners have been used for acquisition, which could have introduced systematic variability in the measurements between the centers. The different CMR scanners also precluded comparison of absolute quantification of T1 and T2 mapping, for which instead normalized values have been reported. Also, T2*-weighted CMR was not part of the CMR protocol, but is currently the method of choice for detecting IMH, due to its sensitivity for detecting distortion of magnetic field homogeneity caused by iron deposition and hemoglobin breakdown products [23,24]. Last, not all patients in the current study underwent follow up CMR, which underscores the need for larger studies or aggregation of individual patient data of completed clinical trials to increase sample size.

## 5 Conclusion

Although STEMI patients who received post-PCI sonothrombolysis had similar infarct size when compared to control patients, they may have more viability within this infarct zone, which results in improved LV systolic function at follow up. Furthermore, sonothrombolysis in this setting was not associated with higher incidence of IMH or MVO compared to the control group. Further investigation is warranted to explore the specific effects of post-PCI sonothrombolysis on the contractility of myocardial segments within both the infarct zone and the remote myocardial regions.

## CRediT authorship contribution statement

**Soufiane El Kadi:** Writing – review & editing, Writing – original draft, Methodology, Investigation, Formal analysis, Data curation, Conceptualization. **Shouqiang Li:** Writing – review & editing, Investigation, Conceptualization. **Chad Hovseth:** Writing – review & editing, Methodology, Formal analysis. **Luuk H.G.A. Hopman:** Writing – review & editing, Methodology, Formal analysis. **Mariëlle C. van de Veerdonk:** Writing – review & editing, Methodology, Formal analysis. **Niels J.W. Verouden:** Formal analysis. **Feng Xie:** Formal analysis. **Albert C. van Rossum:** Writing – review & editing. **Otto Kamp:** Writing – review & editing, Writing – original draft, Investigation, Conceptualization. **Thomas R. Porter:** Writing – review & editing, Writing – original draft, Supervision, Methodology, Conceptualization.

## Funding

This study received funding from the Theodore F. Hubbard foundation and Lantheus Medical Imaging.

## Declaration of competing interest

The authors declare the following financial interests/personal relationships which may be considered as potential competing interests: T.R. Porter receives consultant fees from Lantheus Medical Imaging, Inc., and research equipment support from Philips Research North America. The other authors confirm that they have no competing interests.
